# A minimal double quantum dot

**DOI:** 10.1038/s41598-017-10814-z

**Published:** 2017-09-07

**Authors:** Hao Zheng, Junyi Zhang, Richard Berndt

**Affiliations:** 1 School of Physics and Astronomy, Shanghai Jiao Tong University, Shanghai, 200240 China; 2 Institut für Experimentelle und Angewandte Physik, Christian-Albrechts-Universität zu Kiel, D-24098 Kiel, Germany; 3 Collaborative Innovation Center of Advanced Microstructures, Nanjing, 210093 China; 4 Department of Physics, Princeton University, Princeton, New Jersey 08544 USA

## Abstract

Double quantum dots (DQDs) are a versatile platform for solid-state physics, quantum computation and nanotechnology. The micro-fabrication techniques commonly used to fabricate DQDs are difficult to extend to the atomic scale. Using an alternative approach, which relies on scanning tunneling microscopy and spectroscopy, we prepared a minimal DQD in a wide band-gap semiconductor matrix. It is comprised of a pair of strongly coupled donor atoms that can each be doubly charged. The donor excitation diagram of this system mimicks the charge stability diagram observed in transport measurements of DQDs. We furthermore illustrate how the charge and spin degrees of freedom of the minimal DQD may be used to obtain a single quantum bit and to prepare a Bell state. The results open an intriguing perspective for quantum electronics with atomic-scale structures.

## Introduction

**Figure 1:**
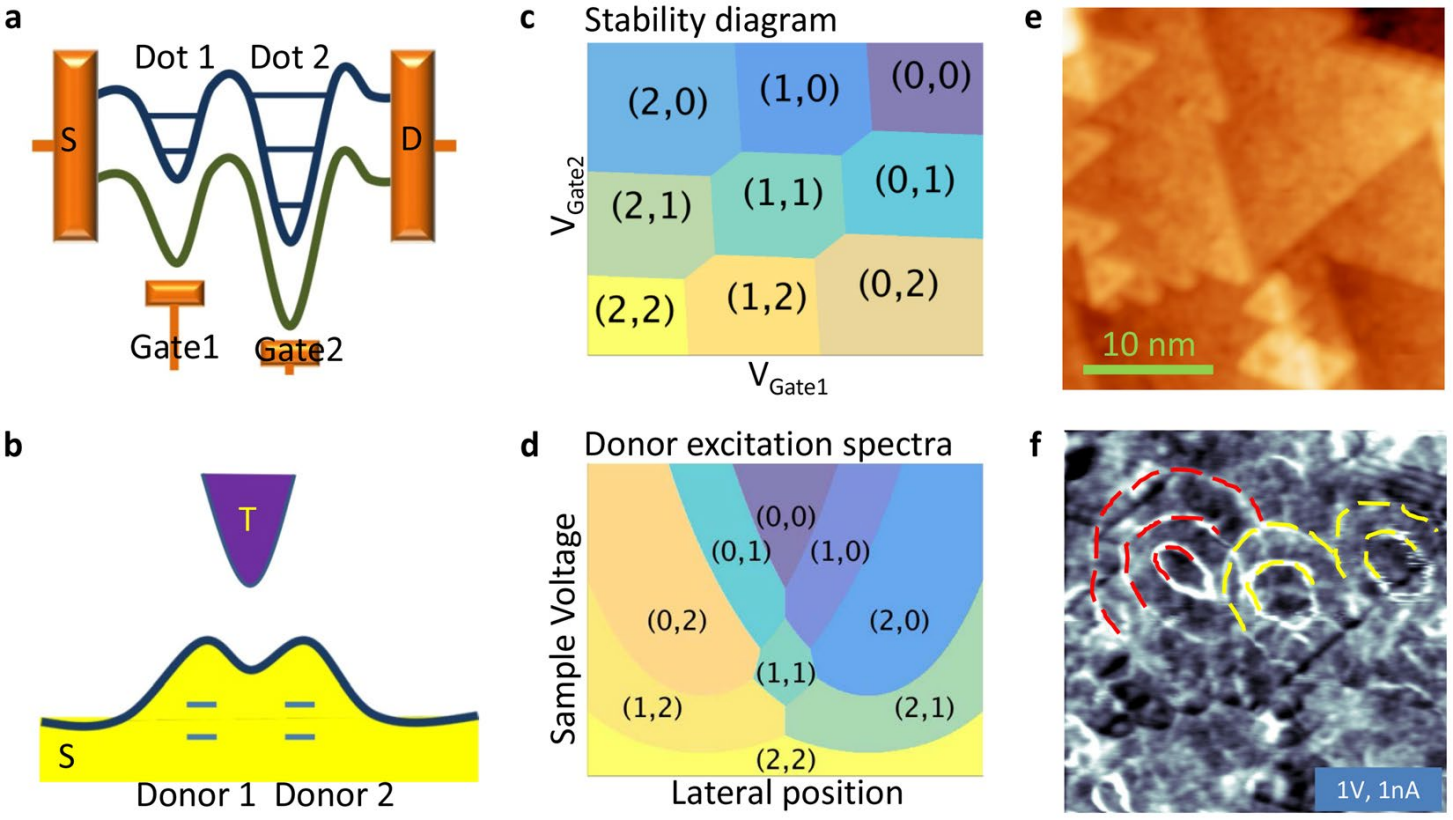
Realizing a minimal double quantum dot. (**a**) A sketch depicting the energy diagram of a micro-fabricated DQD, usually at the nanometer scale. The conductance is measured between the source (S) and drain (D) leads. The local potential together with the quantized energy levels inside of each single QD (Dot 1 and Dot 2) can be tuned by the two gate electrodes, which are marked as Gate 1 and 2 in the sketch. (**b**) A schematic of a double-donor atom dimer in a STM junction, where it serves as a minimal DQD. When a tip (T) and a semiconducting sample (S) form a tunneling contact, the work function difference between the two materials induces a bending of the bands of the sample underneath the tip. The applied bias voltage further alters the degree of band bending. Using the bias and the lateral tip-donor distance, the levels (two blue lines) of each donor can be tuned. Consequently, the excited state (the occupation number) of each donor atom can be controlled, and the conductance of a coupled atomically DQD can be measured. (**c**) Sketch of a typical stability diagram of a strongly-coupled DQD measured by electronic transport experiment. The charge states of each QD is controlled by the potentials of a gate electrode. *n*_1_ (*n*_2_) is the occupation number of QD1 (QD2). (**d**) Sketch of a donor excitation diagram of a dimer of double-donors in a strongly-coupled regime measured by STM. The combination of the bias voltage and the lateral tip position has the same function as the two gate voltages in (**c**) which makes this spectroscopic map equivalent to (**c)**. (**e**) Constant-current STM image (1 V, 1 nA, 5 K) of a clean ZnO(0001) surface area. Large terraces with triangular islands are resolved. (**f**) *dI*/*dV* map simultaneously acquired with (**e**). Yellow dotted lines mark the positions of two set of concentric rings, which originate from two weakly coupled subsurface double-donor atoms. Red lines indicate a set of three concentric rings, which are induced by a dimer of strongly coupled double-donors. ^*a*^The latter case realizes a minimal DQD. ^*a*^In principle, a strongly-coupled double-donor can lead to four rings like in Fig. 2a. The fourth ring is too weak in the present case to be discernible amidst various irregularities of the ZnO crystal.

Quantum dots (QDs) are artificial nanometer scale structures in which quantum confinement causes the formation of discrete energy levels from continuous electronic bands of a solid. A single QD may be considered as an artificial atom, while a double QD (DQD) can be viewed as a molecule^1^. Depending on the strength of the coupling between the QDs, DQDs may be categorized as weakly-coupled or strongly-coupled^1^. Strongly-coupled DQDs have attracted much research attention, due to their fundamental properties and their significant applications. The Coulomb staircase, the spin blockade effect, the use as a single spin quantum bit, the realization of a micromaser are just a few of many examples^1–4^. A typical DQD involves source and drain leads, which are coupled to the DQD with tunneling contacts, and two gate electrodes, which individually control the local potential of each QD (Fig. 1a). By varying both gate voltages and measuring the source-drain conductance, the charge states of the DQD may be determined and displayed in the so-called stability diagram (Fig. 1c). The stability diagram may be used to extract the coupling strength and also reveals additional key parameters, such as the on-site binding energy and Coulomb interaction.

To further shrink the dimensions of DQDs to the atomic scale scanning tunneling microscopy and spectroscopy (STM/S) is an obvious choice. Indeed, STM has successfully been used to investigate single impurities in semiconductors like Si or GaAs^5^. Examples of recent achievements are the controlled switching of the charge state of a single impurity, the manipulation of individual donor binding energies, the magnetization of individual dopant, and the observation of a valley interference effect in single dopants^6–11^. However, the lack of a gate electrode is a significant drawback of STM in transport measurements, which are essential for studies of QDs. Here, we have present a data acquisition and analysis method that enables measurements of the stability diagram of atomic-scale DQDs. The DQDs are comprised of dopant dimers.

Monomers and dimers of donors that can carry a single charge have been characterized by STM^6–9, 12–14^. However, only donors that can be multiply charged are suitable for implementing a DQD. Therefore, we used the third generation semiconductor material ZnO^15^. In ZnO double-donors, which can be charged with up to two electrons, are available^16, 17^. From measured donor excitation diagrams (Fig. 1d) we found that a dimer of double-donors can be viewed as a DQD. In order to obtain such data, a set of differential conductance spectra (*dI*/*dV*) measured along a line crossing two neighboring double-donor atoms is required. The spectra are then represented as a two-dimensional map that displays the conductance vs. the bias voltage and the lateral tip position. The ionization of the donors leads to a peak in *dI*/*dV* that evolves with the position of the tip and separates the map into several regions. The distinct areas of such a map correspond to different occupation numbers (*n*_1_, *n*_2_) of the two donors, where *n*_1_ (*n*_2_) is the number of electrons on the left (right) dot.

The donor excitation diagrams from our STM experiment closely resemble the stability diagrams obtained in transport measurements on DQDs. In other words, pairs of double-donors in ZnO represent minimal DQDs, with each QD involving a single donor and the surrounding ZnO lattice.

The clean ZnO(0001) surface displays terraces with triangular islands of adatoms and vacancies^18, 19^ as shown in Fig. 1e. Despite the rough surface morphology, the *dI*/*dV* map exhibits sharp rings, similar to what has been observed on other materials, such as GaAs, Bi_2_Se_3_ and MoSe_2_. These rings are due to the ionization of subsurface dopant atoms^6, 20, 21^. Importantly, ZnO features concentric double and multiple rings, which result from double or multiple charging of a donor or donor dimer at their centre.

**Figure 2:**
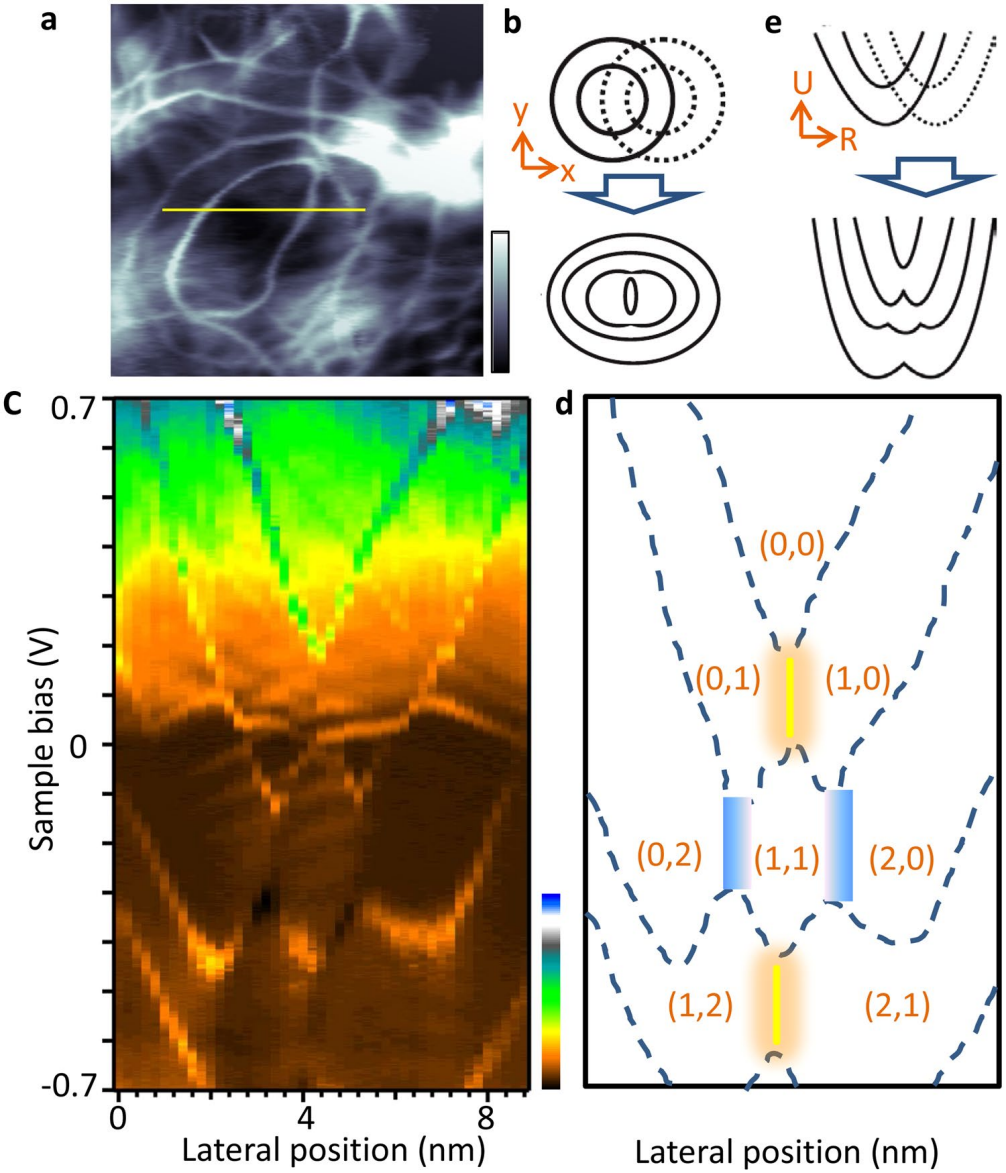
A symmetric minimal DQD. (**a**) 15 × 15 nm^2^ size *dI*/*dV* map, which was acquired at 0.7 V and 1 nA. A strongly coupled DQD can be identified by the presence of multiple (instead of two) concentric ionization rings. When the two subsurface donors are located at the same depth, the DQD exhibits symmetric spectroscopic features. (**b**) Sketch displaying one pair of identical double ionization rings in the weakly (upper panel) and strongly coupled (lower panel) regimes. The lower panel is consistent with (**a)**. (**c**) Measured donor excitation diagram. The diagram was generated from 45 *dI*/*dV* spectra displayed in the parameter space spanned by the voltage and the tip position. The spectra were measured along the yellow line in (**a)**. Each spectrum was obtained at the set point of 0.7 V and 1 nA. From the fits described in Supplementary Fig. 1, the distance between the donor atoms is estimated to 6 nm. (**d**) Sketch of (**c)** highlighting important features of the data. The dotted lines reproduce the charge excitation lines in (**c**) and separate regions with different charge occupations (*n*_1_, *n*_2_). Yellow and blue areas are discussed in Fig. 4. One may note that there are some additional features in the (0,2) and (1,1) area of (**c**). They are due to vibrational excitations as discussed in ref. 17. (**e)** Schematic donor excitation diagram from a symmetric DQD in a weakly (upper panel) and strongly coupled (lower panel) regime. The measured spectra represent examples of the strongly-coupled case.

In Fig. 1f, two neighboring elliptical double-rings (yellow lines) are observed. Their centers are ≈10 nm apart and their overlapping contours apparently do not affect each other. These properties are expected from weakly coupled QDs. In addition, the spectroscopic map displays a system exhibiting three concentric rings (red lines). Cross-sectional profiles of the multiple-ring system (Fig. 2a and c), reveal a donor excitation diagram that is typical of a strongly coupled DQD.

**Figure 3:**
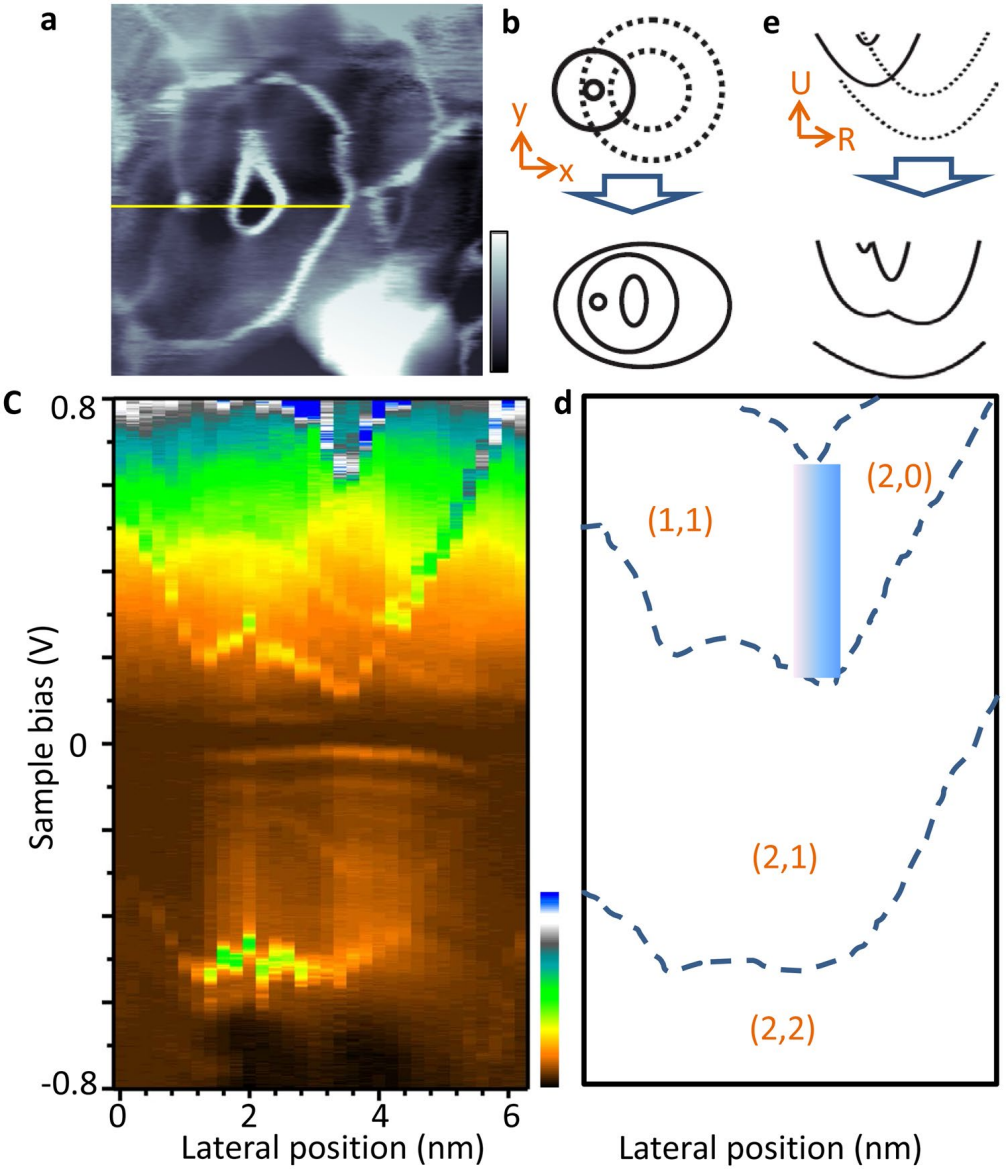
An asymmetric minimal DQD. (**a**) *dI*/*dV* map of two subsurface donors, buried at different depths, exhibiting asymmetric spectroscopic features. (**b**) Ssketches of a pair of distinct double ionization rings in the weakly (upper panel) and strongly coupled (lower panel) regimes. (**a)** Is identified as a strongly-coupled case. (**c**) Measured donor excitation diagram. The diagram contains 32 *dI*/*dV* spectra, measured along the yellow line in (**a**) with a set point of 0.8 V and 1 nA. (**d**) Schematic diagram of (**c)**. Dotted lines reproduce the charge excitation lines in **c**, and separate areas with distinct occupation numbers as indicated. The blue area will be discussed in Fig. 4. (**e**) Schematic donor excitation diagram of an asymmetric DQD in the weakly (upper panel) and strongly coupled (lower panel) regimes. The measured spectra are consistent with strongly-coupled case.

**Figure 4:**
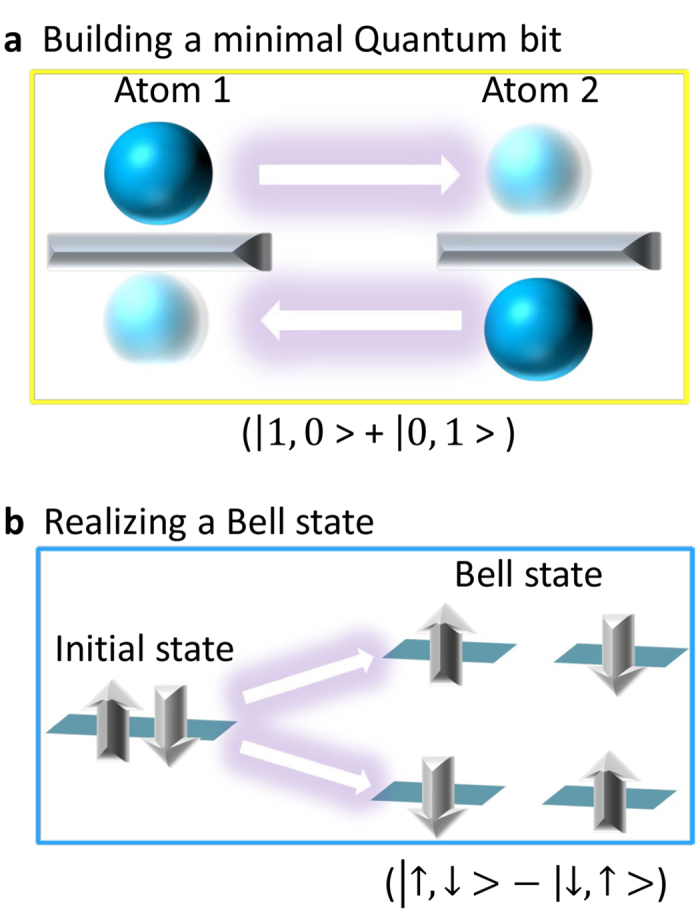
Applications of a minimal DQD. By shining microwave radiation with proper wavelength into the STM junction, the charge and spin states of a DQD may be coherently manipulated and entangled quantum states may thus be realized at the atomic scale. (**a**) STM tip position and voltage are stabilized at the boundary between (0,1) and (1,0) as well as the boundary between (1,2) and (2,1), which are the blue areas in Figs 2d and 3d, the coherent coupling between the two quantum states from the two donor atoms gives rise to a (|0, 1〉 + |1, 0〉) qubit. The solid (transparent) sphere represents the occupied (empty) charge states of a donor. (**b)** depicts a process leading toward a Bell state. It requires that the STM tip initially stabilizes (2,0) or (0,2) states (yellow regions in Fig. 2d), meaning that one donor is free of electrons and the other is occupied by two electrons with opposite spins. When the DQD is adiabatically tuned into the (1,1) state with microwave radiation, the final state realizes a Bell state $|{{\rm{\Psi }}}_{B}^{-}\rangle =\frac{1}{\sqrt{2}}(|\uparrow ,\downarrow \rangle -|\downarrow ,\uparrow \rangle )$.

Previous works have shown that the binding energy (ionization threshold) of a subsurface donor is largely determined by its depth underneath the surface^22, 23^. In other words, donor atoms at the same depth exhibit identical donor excitation diagram and ionization rings in real-space *dI*/*dV* maps. Therefore, a pair of donors at identical depths is expected to generate a symmetric DQD while different depths introduce a degree of asymmetry. The capability of producing both symmetric and asymmetric DQDs is essential for application as a quantum light source^24, 25^. In the STM/S data of Figs 2 and 3, we indeed observed both types of DQDs. Using the evolution from a weakly-coupled DQD to the strongly-coupled case (Figs 2e and 3e), we can identify charge occupation numbers (*n*_1_, *n*_2_). As shown in Figs 3d and 4d, the coherent superposition of particular DQD charge states, *e.g*. at the boundary between two areas with different (*n*_1_, *n*_2_), exhibits potential application to quantum electronics.

To describe the dominant features of the donor excitation diagram and to reveal the potential application of our minimal DQD we build an analytic model. The total Hamiltonian is given by

1 $$\begin{array}{rcl}H & = & {H}_{1}+{H}_{2}+{H}_{C}={H}_{1}+{H}_{2}+{H}_{T}+{H}_{m}+{H}_{s}\\ {H}_{1} & = & {n}_{1}({\epsilon }_{d}-{\mu }_{t1})+U{n}_{1}({n}_{1}-1);{H}_{2}={n}_{2}({\varepsilon }_{d}-{\mu }_{t2})+U{n}_{2}({n}_{2}-1)\\ {H}_{T} & = & -{t}_{12}{c}_{1}^{\dagger }{c}_{2}+h.c\mathrm{.}\\ {H}_{m} & = & {U}_{m}{n}_{1}{n}_{2}.\\ {H}_{s} & = & -{n}_{1}{\gamma }_{12}e(V-{V}_{F}).-{n}_{2}{\gamma }_{21}e(V-{V}_{F})\end{array}$$

*H*_1_ (*H*_2_) is the Hubbard Hamiltonian for the left (right) donor atom. *H*_*C*_, the inter-donor coupling, is comprised of three terms. *H*_*T*_ describes coherent tunneling, *H*_*m*_ is the Coulomb interaction between two donors, and *H*_*s*_ represents the indirect chemical potential shift. In these equations, ${\epsilon }_{d}$ is the first electron’s on-site binding energy; *μ*_*t*_ describes the effective local chemical potential due to tip-induced band-bending (TIBB); *U* stands for the on-site Coulomb repulsion between two electrons at same donor; *t*_12_ is the coherent coupling strength; *U*_*m*_ is an inter-atom Coulomb repulsion; *V*_*F*_ denotes the flat band voltage; *V* is the bias of the tip; *γ*_*ij*_ is the TIBB of site *i* induced by TIBB of site *j* via the coupling between the donor atoms.

In our experiment, the two sites of the DQD are spatially separated (about 6.5 nm), direct coherent tunneling is suppressed by the barrier, so *H*_*T*_ is negligible. The physics of the DQD can be understood in a semi-classical picture. In this regime, *n*_1_ and *n*_2_ are both good quantum numbers, and the Hamiltonian can be directly diagonalized. By taking into account the position-dependent TIBB (see details in Supplementary Note 1 and Supplementary Fig. 1), we are able to simulate the symmetric DQD donor excitation diagram and deduce all key parameters as listed in Table 1. Specifically, we obtained ${\epsilon }_{d}$ = −200 meV and *U* ≅ 90 meV for individual donors and *U*_*m*_ ≅ 30 meV for the inter-donor coupling.

**Table 1:** Microscopic parameters deduced from experiments.

Data set	${{\boldsymbol{\epsilon }}}_{{\boldsymbol{d}}}$(meV)	*U*(meV)	*V*_*F*_(meV)	*β*_0_	*σ*(nm)	*U*_*m*_(meV)	*γ*	*d*(nm)
DQD1	−200	90	−920	−0.27	5.76	30	0.09	6.12
DQD2	−200	95	−850	−0.27	5.4	40	0.09	7.02

${\epsilon }_{d}$ is the on-site electron binding energy; *U* is the on-site Coulomb repulsion between the two electrons; *V*_*F*_ is the flat band voltage. The shape of the TIBB is described by a squared Lorentzian function. *σ* is the width of a squared Lorentzian and *β*_0_ is the height of the squared Lorentzian. *U*_*m*_ is inter-atom Coulomb interaction; *d* is the lateral distance between two donor atoms.

By shining microwave radiation with a suitable wavelength into the STM junction^26, 27^, coherent tunneling (microwave assisted tunneling) between the two donor atoms may be induced. In such a scenario, it appears possible to build a quantum bit and a Bell state in our minimal DQD by utilizing its charge and spin degrees of freedom. To obtain a quantum bit, the DQD system is first initialized to an occupation (0,1), where the donors carry 0 and 1 electron, respectively. Next, the microwave energy is tuned close to resonance with the (0,1)–(1,0) transition and a coherent coupling between these two states is built up. This process turns the minimal DQD into a minimal single solid-state charge qubit, similar to proposals based on a micro-fabricated QD with larger size^28^. To prepare a Bell state, the system is initialized to the (0,2) state. The double donors in our ZnO samples most likely are interstitial Zn atoms^16, 17^. When the donor carries two electrons, they occupy the Zn 4*s* orbital, thus forming a spin singlet state. Moving one electron to the other donor atom thus produces a fourfold degenerate (1,1) state. Since the coherent tunneling conserves the total spin *S* ^2^ and its *z*-component *S*_*z*_, only the spin singlet (1,1) state, *i.e*. the Bell state $|{{\rm{\Psi }}}_{B}^{-}\rangle =\frac{1}{\sqrt{2}}(|\uparrow ,\downarrow \rangle -|\downarrow ,\uparrow \rangle )$, couples to the singlet (0,2) state. By applying the stimulated Raman adiabatic passage protocol (see details in Supplementary Note 2) to the (0,2)–(1,1) transition, the DQD is adiabatically driven from spin-singlet-(0,2) to a spin-singlet-(1,1) state^29, 30^. The destination of our DQD is thus in the Bell state $|{{\rm{\Psi }}}_{B}^{-}\rangle$.

In summary, we have experimentally realized a strongly-coupled DQD which is comprised of a dimer of donor atoms. The electronic levels of the individual donors depend on the depth below the surface, which can break the symmetry between the donors of a pair. As recently demonstrated the depth of an individual donor in ZnO can be manipulated^23^ and consequently the electron transport properties of the proposed DQD may be controlled. An analytical model is capable of reproducing the electronic states of the system. We propose that microwave radiation may be used to prepare interesting entangled quantum states of this minimal DQD.

For the time being, STM-based techniques enable probing these electronic properties. Defect densities in ZnO crystals are high and the experiments require a time-consuming search for a suitable sample area. With improved crystal quality, however, it may become possible to more routinely obtain these DQD presented above.

## Methods

### Experiment

All experiments were performed in an ultra-high vacuum system equipped with a home-built STM operated at 5 Kelvin. Single-crystalline ZnO(0001) was prepared by cycles of Ar^+^ bombardment and high temperature annealing. Au tips were cut from a polycrystalline wire and *in situ* annealed prior to transfer to the STM. STM imaging was performed in a constant-current mode with the bias voltage being applied to the sample. A sinusoidal voltage modulation of 10 mV_rms_ and a lock-in amplifier were employed to measure *dI*/*dV* spectra.

### Theory

We build a total Hamiltonian to describe the strongly-coupled donor dimer. In the semi-classical regime, the Hamiltonian can be directly diagonalized enabling a simulation of the predominant features in the experimental data. In the quantum regime, we used two effective total Hamiltonians to describe the charge quantum bit and spin Bell state. Details of the theory are provided in the Supplementary Figs 1 and 2 and Supplementary Notes 1 and 2.

## Electronic supplementary material

Supplementary Information
