# Supplementary material for: A minimal double quantum dot
Source: Sci Rep. 2017 Sep 7;7:10764. doi: 10.1038/s41598-017-10814-z (PMC5589847; doi:10.1038/s41598-017-10814-z)
Supplement: Supplementary file 1 — Supplementary Information [file 41598_2017_10814_MOESM1_ESM.pdf]

A minimal double quantum dot

Hao Zheng,[1,2,3] Junyi Zhang,[4] and Richard Berndt[2]

[1]*Department of Physics and Astronomy,*
Shanghai Jiao Tong University, Shanghai 200240, China

[2]*Institut für Experimentelle und Angewandte Physik,*
Christian-Albrechts-Universität zu Kiel, D-24098 Kiel, Germany

[3]*Collaborative Innovation Center of Advanced Microstructures, Nanjing 210093, China*

[4]*Department of Physics, Princeton University,*
Princeton, New Jersey 08544, USA

A. Supplementary Note

1. *Semiclassical regime– Simulation of experimental donor excitation diagram*

As stated in the main text, the total Hamiltonian (H) describing our double-donor dimer is

$$\begin{aligned} H &= H_1 + H_2 + H_C = H_1 + H_2 + H_m + H_s + H_T \\ H_1 &= n_1(\epsilon_d - \mu_{t1}) + U n_1(n_1 - 1); H_2 = n_2(\epsilon_d - \mu_{t2}) + U n_2(n_2 - 1) \\ H_m &= U_m n_1 n_2 \\ H_s &= -n_1\gamma_{12}e(V - V_F) - n_2\gamma_{21}e(V - V_F). \\ H_T &= -t_{12}c_1^\dagger c_2 + h.c. \end{aligned} \tag{1}$$

H_1 and H_2 are the Hubbard Hamiltonians for the left and right donor atom, respectively. H_C describes the inter-donor coupling, which comprises three items: 1) H_m, the Coulomb interaction between two donors; 2) H_s, the indirect chemical potential shift; 3) H_T, the coherent tunneling. The indirect chemical potential shift reflects the effect that the TIBB at one donor may induce a redistribution of the background charge around it and consequently change the local chemical potential at another donor.

In our STM experiment, the two donor atoms are spatially separated about 6.5nm, direct coherent tunneling is suppressed by the barrier, so the coherent tunneling term H_T is negligible. (n_1, n_2) are good quantum numbers and the system is in the semi-classical regime. To simulate our donor excitation diagram, position-dependent tip-induced band bending effect should be taken into account. This effect is mainly characterized by the shifted effective local chemical potential that is assumed to be proportional to $V - V_F$ (V_F is the flat band voltage). It is modified by a tip–sample–distance–dependent factor $\beta(x)$ describing the effect of the tip, bulk screening and the environment. It has been found that a squared Lorentzian factor

$$\beta(x) = \beta_0 \left[\frac{\sigma^2}{\sigma^2 + x^2}\right]^2 \tag{2}$$

describes the experimental observations well. Therefore, the chemical potential μ_t in Eqn. 1 becomes voltage (V) and position (x) dependent.

$$\mu_t = e\beta(x)(V - V_F). \tag{3}$$

To simulate a symmetric DQD, we set $\gamma_{ij} = \gamma\beta_j$, where β_j is the TIBB form factor of site j and γ is a small dimensionless constant (about 0.1) describing the the strength of the inter-donor chemical potential shift due to TIBB. Figure S1 displays fits to two strongly-coupled symmetric DQD excitation diagrams from the experiments. The fits serve to derive the key parameters in Table 1 of the main text, such as the on-site binding energy ϵ_d , the on-site Coulomb repulsion U and inter-site Coulomb interaction U_m.

2. Quantum regime — Realization of entangled quantum states

Next we discuss the model in the presence of coherent tunneling, *i.e.* $t_{12} \neq 0$. The occupation numbers of each site are no longer good quantum numbers, the semiclassical picture breaks down, and the system can be in states that are superpositions of semiclassical states. For simplicity, we assume that the microwave used for generating coherent tunneling couples to the donor pair through the electric dipole moment. Therefore, t_{ij} is independent of the spin. We also assume that the frequency of the microwave is near-resonant to the transitions between the relevant states. The effective Hamiltonian for the light-coupled transitions can be written in a simple form with the rotating-wave approximation[1,2]. This approximation more clearly reveals the relevant physics as it leads to closed form solutions an avoids tedious mathematics.

To initialize the system to a desired state, *e.g.* in $(0,1)$ or $(0,2)$, the tip is moved to a suitable position and a bias voltage is applied.

1) Starting from the $(0,1)$ state, in order to operate the donor pair as a qubit, coherent tunneling must be enabled by adjusting the microwave frequency close to resonance with the $(0,1)-(1,0)$ transition. The effective total Hamiltonian of the two–level system is given by

$$H_{TL} = \frac{\hbar}{2}\begin{pmatrix} \delta & \Omega \\ \Omega^* & -\delta \end{pmatrix}, \tag{4}$$

where δ is often called detuning and Ω is denoted Rabi frequency in atomic physics. Here the detuning is defined as $\hbar\delta = E_{(0,1)} + \hbar\omega_L - E_{(1,0)}$, where ω_L is the frequency of the microwave. The Rabi frequency Ω is proportional to the complex amplitude of the microwave. The qubit may be manipulated by tuning the amplitude and frequency of the microwaves.

In contrast to atomic or molecular systems, the spontaneous decay of the quantum entanglement in our DQD system is weak. The main source of decoherence here is thermal agitation. However, at 5 K, the temperature of the experiment, the thermal energy is much smaller than the smallest of all interactions, namely the inter-site Coulomb interaction $U_m \approx 30$ meV, which was determined from the spectroscopic data. Therefore, the single charge qubit implemented in the DQD should be stable.

2) The DQD may also be used to prepare a Bell state. We assume that all conditions identical to those in the previous example, except that the system is initialized to the (0,2) state, where two electrons occupy the same orbital of the same donor, thus forming a spin singlet. Then the near-resonant microwave couples the (0,2) state to the $(1,1)$ state that is fourfold degenerate due to the spin degrees of freedom. The effective total Hamiltonian is

$$H_B = \frac{\hbar}{2}\begin{pmatrix} 0 & \Omega^* & 0 \\ \Omega & 2\delta & \Omega \\ 0 & \Omega^* & 0 \end{pmatrix} . \qquad (5)$$

Since microwave assisted tunneling conserves angular momentum, only the $J_z = 0$ states are coupled to $(0, 2)$ state. The $|\uparrow,\downarrow\rangle + |\downarrow,\uparrow\rangle$–state is decoupled as a dark state. By adiabatically ramping up the detuning from the far red to the far blue, one may prepare the Bell state $|\uparrow,\downarrow\rangle - |\downarrow,\uparrow\rangle$ from the initial singlet state. The practical protocol of this process is often referred to as stimulated adiabatic Raman passage (STIRAP) in atomic physics. The level diagram of the STIRAP and the corresponding fidelity $|\langle B|\psi\rangle|^2$ are shown in Fig. S2.

B. Supplementary Figures

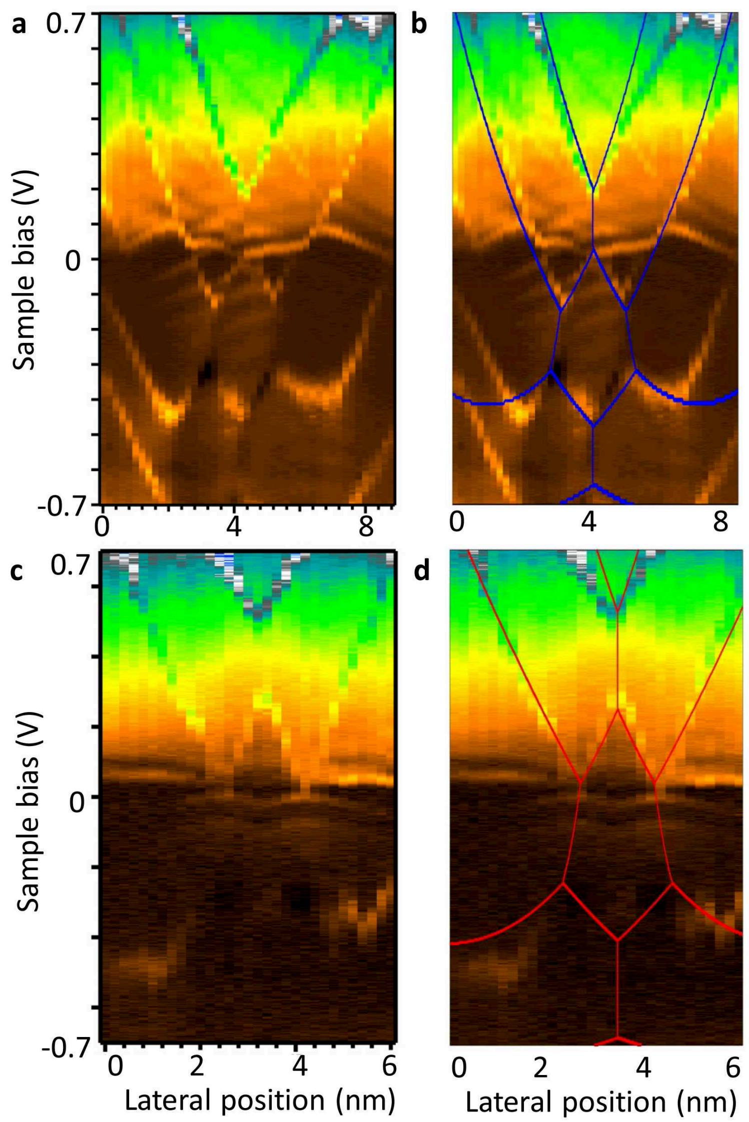

FIG. 1: **Simulated and experimental donor excitation diagrams of two symmetric DQDs.** **a** and **c**, show two donor excitation diagram acquired from two double-donor dimers on the same ZnO(0001) sample. In **b** and **d**, simulated curves from the model are superimposed on the experimental data.

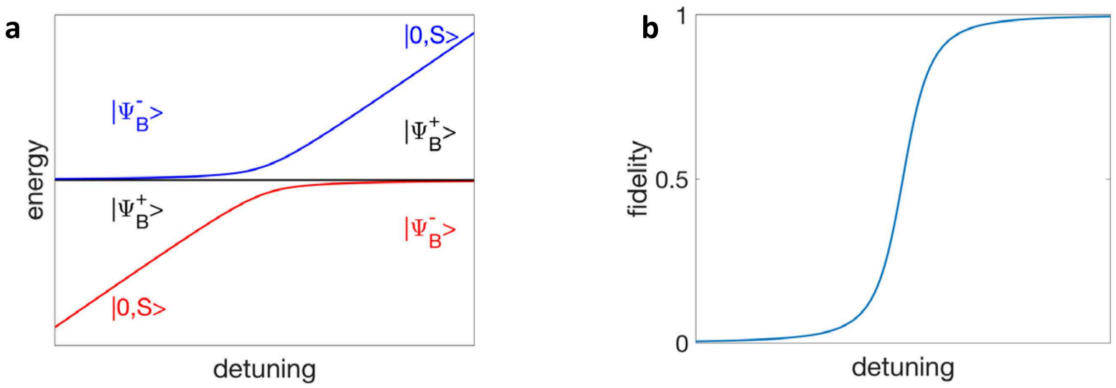

FIG. 2: **Stimulated Adiabatic Raman Passage.** **a**, Energy spectrum of Hamiltonian H_B versus detuning. When the microwave is detuned far to the red, the ground state is a singlet of two electrons on the same site $|0, S\rangle$. Detuning to the blue leads to the Bell state $|\Psi_B^-\rangle$. **b**, fidelity of the ground state $|\psi\rangle$ of the Hamiltonian H_B versus detuning. The fidelity is defined as $\mathcal{F} = |\langle\psi|\Psi_B^-\rangle|^2$, which reflects the overlap of the ground state and the targeted Bell state.

[1] Haroche, S. & Raimond, J.-M., *Exploring the Quantum: Atoms, Cavities and Photons*, pp 152, Chap 3, Eq. (3.167), Oxford University Press 2006.

[2] Stafford, C. A. & Wingreen, N. S., Resonant Photon-Assisted Tunneling through a Double Quantum Dot: An Electron Pump from Spatial Rabi Oscillations. *Phys. Rev. Lett.* **76**, 1916 (1996).
